# Postpartum Physical Activity and Weight Retention within One Year: A Prospective Cohort Study in Vietnam

**DOI:** 10.3390/ijerph17031105

**Published:** 2020-02-10

**Authors:** Anh Vo Van Ha, Yun Zhao, Colin W. Binns, Ngoc Minh Pham, Phung Thi Hoang Nguyen, Cong Luat Nguyen, Tan Khac Chu, Andy H. Lee

**Affiliations:** 1 School of Public Health, Curtin University, Perth, WA 6845, Australia; vovananh.ha@postgrad.curtin.edu.au (A.V.V.H.);; 2 Faculty of Public Health, Pham Ngoc Thach University of Medicine, Ho Chi Minh City 700000, Vietnam; 3 Faculty of Public Health, Thai Nguyen University of Medicine and Pharmacy, Thai Nguyen 250000, Vietnam; 4 Faculty of Public Health, University of Medicine and Pharmacy at Ho Chi Minh City, Ho Chi Minh City 700000, Vietnam; 5 National Institute of Hygiene and Epidemiology, Hanoi 100000, Vietnam; 6 Faculty of Public Health, Hai Phong University of Medicine and Pharmacy, Hai Phong 180000, Vietnam

**Keywords:** physical activity, pregnancy, body weight, postpartum weight retention, cohort study, Vietnam

## Abstract

After delivery, mothers are encouraged to increase physical activity (PA) gradually to regulate body weight; however, data on PA in relation to postpartum weight retention remains scarce, particularly among Asian women. In a cohort of 1617 Vietnamese mothers, we investigated the prospective association between habitual PA exposures at 3-month postpartum and weight retention at 6-month and 12-month postpartum. Detailed information on PA intensity and domains was collected from participants using a validated instrument specifically for Vietnamese women. Linear regression analyses and a general linear model for the repeated weight retention measures were used to ascertain the apparent relationships. On average, the participants reported 3.6 (SD 3.9) and 2.6 (SD 3.8) kg weight loss at 6- and 12-month postpartum, respectively. Total and light-intensity PA were inversely associated with the postpartum weight retention (*p* for trend <0.05). Our findings highlight the importance of resuming PA in the early postpartum period as an appropriate weight management strategy.

## 1. Introduction

Maternal overweight and obesity is a public health challenge and contributes to adverse health outcomes for both mother [1,2] and infant [3,4,5]. During the past decade, there has been a greater increase in average body mass index (BMI) among women in Southeast Asia (>1 kg/m^2^) than those residing in higher-income Asian countries (<0.2 kg/m^2^) [6]. A recent meta-analysis estimated that one in five women aged over 15 years was overweight in Southeast Asia, including Vietnam [7].

Excess postpartum weight retention has been linked to long-term maternal–infant outcomes, including metabolism changes in mothers, difficulties in breastfeeding initiation, and infant’s microbiome [8]. Women who did not return to their pre-pregnancy weight and gained weight through one-year postpartum are at increased risk of obesity, insulin resistance, and metabolic syndrome at six-year postpartum [9]. Common risk factors of postpartum weight retention are related to pre-pregnancy and pregnancy periods, including pre-pregnancy BMI, gestational weight gain, and parity [10,11,12].

In 2015, the American College of Obstetricians and Gynecologists advocated physical activity (PA) in the postpartum period to maintain a healthy lifestyle and to manage a healthy weight [13] in mothers. Asian women weigh approximately two kilograms heavier on average at 6- to 12-month postpartum than their pre-pregnancy weight [14,15,16,17]. Since infant care occupies most of a new mother’s time in the postpartum period [18], it remains a challenge for them to engage in sports or exercise programs.

Several studies have examined the relationship between postpartum PA and postpartum weight retention but mostly restricted to Caucasian populations [19]. For example, a study with a prospective cohort of 1213 Australian women reported walking 30 min or more per week in the postpartum period resulted in significant weight loss [20]. Another prospective cohort study of 908 women in the U.S.A. also reported an inverse association between daily 30-min walking and retaining five or more kilograms at 12-month postpartum [21]. These studies focused on the assessment of specific PA intensity (e.g., moderate level) and PA domain (such as sports or exercise) [20,21], while the impact of other intensity (e.g., light-intensity) or domain (e.g., occupational) of PA on postpartum weight retention remains unknown in Asian women [13].

There have been limited investigations on PA for the general population of Vietnam. Nevertheless, a previous study indicated that total PA was inversely associated with BMI, with the mean total PA for women being approximately 50% lower than the overall average, while three-quarters of the 14,706 participants reported engaging in none of the measurable sports/exercise activities [22]. For Vietnamese mothers, recent evidence has suggested that weight gain during pregnancy is related to postpartum weight retention [17]. However, no information is available concerning the role of PA during postpartum period in weight retention. Therefore, the present study aimed to fill the scientific gap by investigating the association between PA (intensity and domain) at 3-month postpartum and weight retention within one-year postpartum using a large cohort of women in Vietnam.

## 2. Methods

### 2.1. Study Design, Setting, and Participants

A multicenter prospective cohort study was conducted in Vietnam. Details of the study design, including sample size calculation and recruitment procedure, have been described elsewhere [23]. Briefly, a total of 2030 singleton pregnant women were recruited and followed up in three cities (Hanoi, Hai Phong, and Ho Chi Minh City) between 2015 and 2017. Eligible women were ≥18 years old, living in the study locations, and not having a serious pre-existing health condition. Of the cohort, 1617 participants (79.7%) were successfully followed up to 12-month postpartum. The study was approved by the Curtin University Human Research Ethics Committee (HR32/2015) and the Hai Phong University of Medicine and Pharmacy Human Research Ethics Committee (No 05/HPUMPRB/2015).

### 2.2. Study Variables

The outcomes were weight retention at 6- and 12-month postpartum, calculated by subtracting the self-reported pre-pregnancy weight from the weight measured at 6- and 12-month postpartum, respectively.

The primary exposure variables were maternal PA during the first three months after giving birth. Such information was collected at 3-month postpartum using a validated Vietnamese version of the Pregnancy Physical Activity Questionnaire [24]. We measured 32 specified activities in four domains, namely, household/caregiving, occupational, sports/exercise, and transportation. The duration, frequency, and intensity of PA were calculated using the Compendium of Physical Activities and then converted to the metabolic equivalent of task hours per week (MET-hour/week). The sum of all activities across domains was defined as the total PA. Light-intensity PA, moderate-intensity PA, and vigorous-intensity PA were defined as 1.5 to <3 METs, 3 to 6 METs, and >6 METs, respectively. Light-intensity PA included activities related to household/caregiving, transportation, and occupational tasks. Moderate-intensity PA was derived from several activities pertaining to household/caregiving, transportation, sports/exercise, and occupational tasks. The variables of total PA, light-intensity, moderate-intensity, household/caregiving, and transportation were further categorized into tertiles (low, medium, and high levels) for subsequent regression analyses. Sports/exercise and occupational activities were dichotomized (yes/no) due to low participation in these activities. Vigorous-intensity PA was not included in the analyses because less than 2% of participants engaged in such high level of activity at 3-month postpartum.

Sociodemographic, pregnancy, and postnatal information was obtained from the interviews and medical records whenever available. These variables included maternal age (<25, 25–34, ≥35 years), education (secondary or lower, high school, college or above), formal employment (yes, no), parity (0, ≥1), mode of delivery (vaginal, Cesarean), and gestational age (<37, ≥37 weeks). Pre-pregnancy BMI and 12-month postpartum BMI were classified as underweight (<18.5 kg/m^2^), normal (18.5–22.9 kg/m^2^), and overweight and obese (≥23 kg/m^2^) following the cut-offs from World Health Organization for Asian populations [25]. We measured total energy intake (Kcal/day) during pregnancy using a validated food frequency questionnaire for Vietnamese adults [26]. Total PA during pregnancy (MET-hour/week) was also quantified at the baseline interview. Gestational weight gain was calculated by subtracting each participant’s self-reported pre-pregnancy weight from her pre-delivery weight, the latter being measured by midwives and documented in hospital medical records [17]. We then classified gestational weight gain as being below (inadequate), within (adequate), and above (excessive), according to the 2009 Institute of Medicine recommendations [27].

### 2.3. Statistical Analysis

Stata 15.1 [28] was used for all statistical analyses. We summarized the socio-demographic and postnatal characteristics of the maternal cohort using descriptive statistics and reported the mean weight retention at 6- and 12-month postpartum, and the mean PA exposures at 3-month postpartum. Comparisons between followed-up participants and dropouts were made using chi-square and *t*-tests.

Separate linear regression analyses were performed to assess the association between weight retention at the two time points and each of the 3-month postpartum PA exposures, namely, total PA, light-intensity PA, moderate-intensity PA, household/caregiving, occupational, sports/exercise, and transportation, accounting for potential confounding factors. These confounding factors were selected based on our literature review with a consideration of the available information in our study; they were maternal age [17,23,24], education [17,24], formal employment [17,24], parity [17,23], mode of delivery [24], pre-pregnancy BMI [17,24], energy intake during pregnancy and total PA during pregnancy [29], gestational age and gestational weight gain [17,24]. The linear regression results were presented in terms of estimated regression coefficients together with their 95% confidence intervals (CI).

The general linear model (GLM) with repeated measures provides analysis of variance when the outcome variable is measured at several time points for each subject. In view of our repeated weight retention measurements at 6- and 12-month postpartum, we further used the GLM with repeated measures to ascertain the relationship between weight retention and the PA exposures, adjusting for the same set of confounders as above. Results from fitting the GLM were presented as a forest plot using Stata’s “coefplot” [30]. Tests for overall linear trends across PA levels were conducted in relation to postpartum weight retention, except for the dichotomous PA variables (sports/exercise and occupational).

PA exposures variables were examined both as continuous and categorical (tertiles) in all the above regression models. To assess the robustness of the associations between PA exposures and weight retentions, we furthermore carried out a subgroup analysis by pre-pregnancy BMI (only for the association between categorical total PA and weight retentions). The results of the subgroup analysis and those based on continuous were reported in a Supplementary file.

In order to meet the regression assumptions; the weight retention outcome variables were checked for normality. The homogeneity of variance of the regression residuals was also assessed for all models. Variance inflation factor (VIF) was used to assess the presence of multicollinearity among the independent variables in the regression analyses.

## 3. Results

The mean age of participants (*n* = 1617) at enrollment was 27.5 (SD 5.2) years. As shown in Table 1, the majority of women were formally employed, multiparous, and had a vaginal delivery with gestational age ≥ 37 weeks. Their mean pre-pregnancy BMI was 20.1 (SD 2.4) kg/m^2^, and the mean gestational weight gain of the cohort was 12.9 (SD 4.0) kg. During pregnancy, total PA during pregnancy was 124.7 (SD 57.3) MET-hour/week, while total energy intake was 2120.8 (SD 746.8) Kcal/day on average (data not shown). There were no significant differences in maternal characteristics and pre-pregnancy BMI between the followed-up participants and dropouts.

The mean weight retention at 6-month was significantly higher than that at 12-month postpartum (1.0 kg; *p* < 0.001). At 6- and 12-month postpartum, 35.6% and 25.6% of the mothers retained 5 kg or more, respectively. The prevalence of overweight/obesity at 12-month postpartum (*n* = 351, 21.7%) was higher than that before pregnancy (*n* = 178, 11%).

Table 2 summarizes the PA exposures of the final cohort at 3-month postpartum. Their mean energy expenditure (total PA) was 150.9 (SD 50.1) MET-hour/week. It is evident that Vietnamese women seldom engaged in sports/exercise (*n* = 356, 21.9%) after delivery. On the other hand, they participated mostly in household/caregiving activities. Indeed, very few women (*n* = 124, 7.6%) returned to formal work within 3 months after giving birth.

Table 3 presents the results from linear regression analyses. All VIFs were <10, suggesting that multicollinearity was not a concern. Total PA and light-intensity PA were inversely associated with weight retention at both 6- and 12-month postpartum, though no significant associations were observed for moderate-intensity PA and subtypes (four domains) of PA.

**Figure 1:**
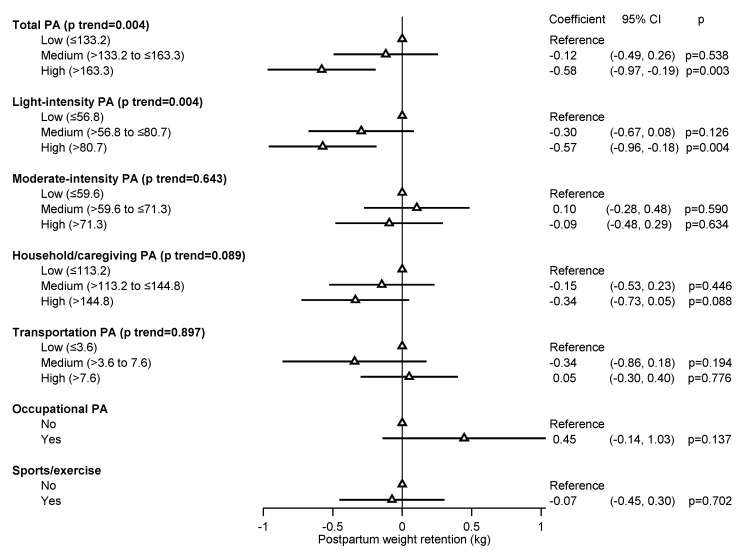
General linear model of one-year postpartum weight retention repeated measures in relation to physical activity at 3-month postpartum. GLM models for each physical activity intensity and domain were adjusted for maternal age at enrolment, education, formal employment, parity, pre-pregnancy BMI, mode of delivery, gestational age, gestational weight gain, total energy intake during pregnancy, and total physical activity during pregnancy; CI, confidence interval; MET, metabolic equivalent of task; PA, physical activity.

Figure 1 displays graphically the results from fitting the GLM to the weight retention repeated measures, which confirms the above results from the separate regression analyses. Vietnamese mothers with high exposure levels of total PA and light-intensity PA experienced a significant reduction in weight retention, but not for engaging in moderate-intensity activities and other domains within one-year postpartum.

The results based on continuous PA exposures variables (see Table S1) are generally in line with those from categorical PA variables, particularly for light-intensity PA. The total PA was found to be inversely associated with weight retentions; however, the associations were only marginally statistically significant. Findings from the sub-group analysis are generally consistent with the findings based on the full sample, particularly for the pre-pregnancy normal weight and underweight groups, which constituted the majority of the examined women (normal weight: *n* = 1018, 63%; underweight: *n* = 421, 26%). For women with pre-pregnancy overweight and obesity (*n* = 178, 11%), total PA appeared to be positively associated with postpartum weight retention, albeit only 72 examined women with overweight and obesity (4.5%) engaged a high level of total PA (see Table S2).

## 4. Discussion

In this prospective cohort of 1617 Vietnamese women, the prevalence of overweight/obesity at 12-month postpartum was double that before pregnancy. This rate is similar to those in other regions. For example, a previous study of 302 African and Dominican women in the U.S.A. observed that 38% of participants were obese seven year after delivery, compared to 22.2% before pregnancy [31]. Another study in 810 Taiwanese women found a substantial increase in overweight and obesity from 18.3% (before pregnancy) to 27.6% (at 6-month postpartum) [32], though no comparable statistics have been reported in Southeast Asia.

The present study represented the first investigation of postpartum weight retention in relation to PA exposures in Vietnam, with comprehensive measurements of postpartum PA in both intensity and domain using a validated instrument for Vietnamese women. Overall, it is evident that a higher PA level (total and light-intensity PA) was associated with less weight retention at 6- to 12-month postpartum.

Our findings are generally consistent with those of previous studies in the postpartum period. A prospective cohort study of 420 Malaysian mothers suggested that increasing postpartum PA (walking, moderate-, and vigorous-intensity activities) could lead to less weight retention [33]. Similarly, another prospective cohort study of 1432 women in Sweden showed that mothers with higher levels of postpartum PA (a combination of occupational and leisure activities) lost more weight when compared to others who were inactive [34].

Light-intensity PA was found to be associated with weight retention within one-year postpartum. A prospective cohort study of 902 women in the U.S.A. similarly reported that walking at least 30 min daily (including sports/exercise and transportation) at 6-month postpartum could reduce weight retention at 12-month postpartum [21]. Although the underlying mechanism remains unclear, it has been suggested that changes in non-exercise activity thermogenesis (including energy expenditure in household/caregiving, transportation, and occupational tasks) may be related to the physiology of body weight regulation [35]. Despite the current PA guidelines recommending moderate-intensity PA in weight management [36], Asian women typically spend less time on moderate-to-vigorous PA during the postpartum period [37]. Nevertheless, our findings indicate that habitual light-intensity PA should be additionally recommended during the early postpartum period for their weight management [36].

We observed no significant associations between moderate-intensity PA, four PA domains, and postpartum weight retention, unlike findings from other countries [20,21,38]. Reasons for the discrepancy are unclear but may be attributed partly to the homogeneity of moderate-intensity PA among mothers engaged in the early postpartum period, that possibly leading to little or no apparent influence of moderate-intensity PA on postpartum weight retention in our study population. Furthermore, the effect of each PA domain on postpartum weight retention might not be discernible when considered separately. During the early postpartum period, Vietnamese mothers are commonly engaged in light-intensity activities (e.g., household/caregiving activities, walking slowly, and sitting), and the sum of such activities contributes substantially to the total PA. Another issue is the instrument used to quantify PA exposure. There has been no similar study in the literature that adopted the same questionnaire as ours to assess habitual PA among postpartum mothers in relation to weight retention.

The Vietnam Employment Law includes entitlement for eligible employees to have up to 6- month paid maternity leave. This policy may contribute to the low number of participants (7.6%) engaging in occupational activities at 3-month postpartum. The apparent lack of association between occupational PA and postpartum weight retention was consistent with a previous prospective cohort study of 550 mothers in the U.S.A. [39]. Furthermore, only a low proportion (21.9%) of Vietnamese women participated in any sports/exercise after giving birth, which may be attributed to busy motherhood for child caring and housework. Similarly, 70% of mothers in Hawaii stated that they were too busy to participate in sports/exercise activities during the postpartum period [40]. In our cohort, mothers who participated in sports/exercise appeared to have less, albeit statistically non-significant, postpartum weight retention. The apparent lack of association concerning sports/exercise and occupational PA was in line with the literature [16,39].

The results based on continuous PA variables further confirmed the inverse associations between PA and postpartum weight retention, particularly for light-intensity PA, which may be the most acceptable and suitable intensity for Asian women in the short-time period after delivery. The categorization of PA variables provided insights on how the specific tertile levels of PA were associated with postpartum weight retentions, which may be useful for implementing practical advice about PA and postpartum weight management.

The subgroup analysis by pre-pregnancy BMI suggested that the inverse association between total PA and postpartum weight retention retained consistently for women with pre-pregnancy normal weight and underweight, but not for those with overweight and obesity. It could be argued that these women were likely to incur an unhealthy lifestyle and perceived more barriers to change such unhealthy behaviors by themselves unless they participate in targeted interventions [41]. Nevertheless, the finding should be interpreted with care due to a very small proportion of women with pre-pregnancy overweight and obesity engaging a high total PA level (*n* = 72, 4.5%) in our sample.

Several strengths and limitations should be acknowledged. A major strength of our prospective cohort study was its large sample size. We also used a validated instrument for Vietnamese mothers to gather information on 32 habitual PA in four domains. Moreover, plausible confounding factors affecting postpartum weight retention had been accounted for in the regression analyses. Although postpartum weight was measured at two different time points, as in most other studies, postpartum weight retention was determined based on the self-reported pre-pregnancy weight. Nonetheless, there was a high level of concordance between self-reported and measured pre-pregnancy body weight [42,43]. Our participants were also recruited from mainly urban and peri-rural areas of three cities in Vietnam. Therefore, the findings cannot be extrapolated to the entire population of Vietnamese women. Given the limited information and available guidelines on the resumption of postpartum PA [19,44], further studies are needed to provide guidance and recommendations on exercise and PA level when women return to work after maternity leave.

## 5. Conclusions

It is essential that mothers resume PA to assist them in returning to a healthy weight in the early postpartum period. Our results suggested that the resumption of daily PA (total PA or light-intensity activities) is associated with less weight retention for Vietnamese mothers within one-year postpartum. Future research is recommended on ways to promote PA and exercise during the postpartum period. This should include studies of mothers who return to employment at 6-month postpartum.

## Figures and Tables

**Table 1:** Characteristics of the maternal cohort of the examined women (*n* = 1617).

Characteristic	Distribution ^a^	Weight Retention at 6-Month Postpartum (kg)	Weight Retention at 12- Month Postpartum (kg)
	3.1 ± 3.8	3.6 ± 3.9	2.6 ± 3.8
**Age at enrollment** (years)	27.5 ± 5.2		
<25	679 (42.0%)	3.8 ± 4.0	2.7 ± 4.0
25–34	769 (47.6%)	3.5 ± 3.7	2.6 ± 3.6
≥35	169 (10.4%)	3.6 ± 3.7	2.4 ± 3.5
**Education**			
Secondary or lower	536 (33.2%)	3.5 ± 3.9	2.7 ± 3.6
High school	416 (25.7%)	3.6 ± 3.8	2.6 ± 4.0
College or above	665 (41.1%)	3.8 ± 3.8	2.6 ± 3.7
**Formal employment**			
No	500 (30.9%)	3.9 ± 3.9	2.6 ± 3.7
Yes	1117 (69.1%)	3.5 ± 3.8	2.6 ± 3.8
**Parity**			
0	627 (38.8%)	3.7 ± 4.0	2.8 ± 4.1
≥1	990 (61.2%)	3.6 ± 3.8	2.5 ± 3.6
**Pre-pregnancy BMI** (kg/m^2^) ^b^	20.1 ± 2.4		
Underweight (<18.5)	421 (26.0%)	4.3 ± 3.4	3.4 ± 3.4
Normal (18.5–22.9)	1018 (63.0%)	3.6 ± 4.0	2.4 ± 3.8
Overweight and Obese (≥23.0)	178 (11.0%)	2.4 ± 3.9	2.1 ± 4.1
**Mode of delivery**			
Vaginal	992 (61.4%)	3.7 ± 3.9	2.5 ± 3.7
Caesarean section	625 (38.6%)	3.5 ± 3.8	2.7 ± 3.9
**Gestational age** (weeks)	38.9 ± 1.3		
<37	67 (4.1%)	2.4 ± 3.7	1.3 ± 3.5
≥37	1550 (95.9%)	3.7 ± 3.9	2.7 ± 3.8
**Gestational weight gain** (kg) ^c^	12.9 ± 4.0		
Inadequate	597 (36.9%)	2.1 ± 3.3	1.3 ± 3.2
Adequate	755 (46.7%)	3.9 ± 3.6	2.9 ± 3.6
Excessive	265 (16.4%)	6.2 ± 4.2	4.8 ± 4.2

^a^ Data are expressed as *n* (%) for categorical variables and mean ± SD for continuous variables; ^b^ Cut-offs from World Health Organization for Asian populations; ^c^ Categorized according to 2009 Institute of Medicine recommendations; MET, metabolic equivalent of task; SD, standard deviation.

**Table 2:** Physical activity of the examined women at 3-month postpartum (*n* = 1617).

Physical Activity (MET-Hour/Week)	Mean ± SD	Minimum	Maximum
**Total**	150.9 ± 50.1	26.6	375.1
**Intensity**			
Light	69.5 ± 31.6	8.2	214.0
Moderate	66.8 ± 27.6	0	253.8
Vigorous	0.03 ± 0.3	0	4.9
**Domain**			
Household/caregiving	130.5 ± 43.9	22.2	298.2
Transportation	7.4 ± 10.7	0	142.6
Sports/exercise	0.2 ± 0.6	0	10.7
Occupational	2.1 ± 9.4	0	111.3

MET, metabolic equivalent of task; SD, standard deviation.

**Table 3:** Linear regression analyses of postpartum weight retention in relation to physical activity exposures (*n* = 1617).

Physical Activity at 3-Month Postpartum (MET-hour/week)		Postpartum Weight Retention (kg) at			
		6-Month ^a^		12-Month ^a^	
	*n* (%)	Coefficient (95% CI)	*p*	Coefficient (95% CI)	*p*
**Total physical activity**		*p* trend = 0.023		*p* trend = 0.003	
Low (≤133.2)	550 (34.0%)	Reference		Reference	
Medium (>133.2 to ≤163.3)	562 (34.8%)	− 0.18 (− 0.59, 0.24)	0.257	− 0.06 (−0.48, 0.36)	0.570
High (>163.3)	505 (31.2%)	− 0.50 (− 0.94, − 0.07)	0.023	− 0.66 (− 1.09, − 0.23)	0.003
**Light-intensity**		*p* trend = 0.005		*p* trend = 0.015	
Low (≤56.8)	553 (34.2%)	Reference		Reference	
Medium (>56.8 to ≤80.7)	543 (33.6%)	− 0.21 (− 0.63, 0.21)	0.325	− 0.38 (− 0.80, 0.04)	0.076
High (>80.7)	521 (32.2%)	− 0.62 (− 1.05, − 0.18)	0.005	− 0.53 (− 0.96, − 0.10)	0.016
**Moderate-intensity**		*p* trend = 0.810		*p* trend = 0.557	
Low (≤59.6)	542 (33.5%)	Reference		Reference	
Medium (>59.6 to ≤71.3)	563 (34.8%)	0.13 (− 0.29, 0.56)	0.536	0.08 (− 0.34, 0.50)	0.723
High (>71.3)	512 (31.7%)	− 0.06 (− 0.48, 0.37)	0.801	− 0.13 (− 0.56, 0.30)	0.543
**Household/caregiving**		*p* trend = 0.117		*p* trend = 0.132	
Low (≤113.2)	541 (33.5%)	Reference		Reference	
Medium (>113.2 to ≤144.8)	553 (34.2%)	− 0.14 (− 0.57, 0.28)	0.500	− 0.15 (− 0.57, 0.27)	0.484
High (>144.8)	523 (32.3%)	− 0.34 (− 0.78, 0.09)	0.116	− 0.33 (− 0.76, 0.10)	0.131
**Transportation**		*p* trend = 0.858		*p* trend = 0.957	
Low (≤3.6)	945 (58.4%)	Reference		Reference	
Medium (>3.6 to ≤7.6)	173 (10.7%)	− 0.11 (− 0.69, 0.46)	0.697	− 0.57 (− 1.14, 0.001)	0.050
High (>7.6)	499 (30.9%)	0.05 (− 0.34, 0.43)	0.818	0.06 (− 0.33, 0.44)	0.638
**Occupational**					
No	1500 (92.4%)	Reference		Reference	
Yes	124 (7.6%)	0.33 (− 0.33, 0.98)	0.327	0.57 (− 0.08, 1.22)	0.088
**Sports/exercise**					
No	1268 (78.1%)	Reference		Reference	
Yes	356 (21.9%)	− 0.22 (− 0.64, 0.20)	0.297	0.08 (− 0.34, 0.49)	0.722

^a^ Separate linear regression models for each physical activity intensity and domain were adjusted for maternal age at enrolment, education, formal employment, parity, pre-pregnancy BMI, mode of delivery, gestational age, gestational weight gain, total energy intake during pregnancy, and total physical activity during pregnancy; CI, confidence interval; MET, metabolic equivalent of task.

## Supplementary Materials

The following are available online at https://www.mdpi.com/1660-4601/17/3/1105/s1, Table S1: Linear regression analyses and GLM with repeated measures of postpartum weight retention in relation to physical activity exposures (*n* = 1617), Table S2: Sub-group analyses: Linear regression analyses and GLM of postpartum weight retention in relation to physical activity exposures (*n* = 1617).
