# Supplementary material for: Postpartum Physical Activity and Weight Retention within One Year: A Prospective Cohort Study in Vietnam
Source: Int J Environ Res Public Health. 2020 Feb 10;17(3):1105. doi: 10.3390/ijerph17031105 (PMC7038097; doi:10.3390/ijerph17031105)
Supplement: Supplementary file 1 [file ijerph-17-01105-s001.pdf]

Table S1. Linear regression analyses and GLM with repeated measures of postpartum weight retention in relation to physical activity exposures (n=1617).

Physical activity at 3-month postpartum (MET-hour/week)	Linear regression analyses				GLM with repeated measures	
	Postpartum weight retention at 6-month [a]		Postpartum weight retention at 12-month [a]		Postpartum weight retention at 6-month [a]	
	**Coefficient (95% CI)**	***p***	**Coefficient (95% CI)**	***p***	**Coefficient (95% CI)**	***p***
Total physical activity	-0.003 (-0.006, 0.001)	0.118	-0.003 (-0.007, 0.00004)	0.053	-0.003 (-0.006, 0.00003)	0.052
Light-intensity	-0.007 (-0.012, -0.001)	0.017	-0.007 (-0.012, -0.001)	0.017	-0.007 (-0.012, -0.002)	0.008
Moderate-intensity	0.001 (-0.005, 0.008)	0.661	-0.002 (-0.008, 0.005)	0.623	-0.0001 (-0.006, 0.006)	0.977
Household/caregiving	-0.003 (-0.007, 0.001)	0.131	-0.004 (-0.008, 0.0004)	0.076	-0.003 (-0.007, 0.0003)	0.069
Transportation	0.0003 (-0.02, 0.02)	0.974	-0.008 (-0.02, 0.01)	0.329	-0.004 (-0.019, 0.011)	0.602
Occupational	0.004 (-0.01, 0.02)	0.688	0.006 (-0.01, 0.02)	0.496	0.01 (-0.01, 0.02)	0.550
Sports/exercise	-0.11 (-0.38, 0.15)	0.406	0.008 (-0.26, 0.27)	0.954	-0.05 (-0.29, 0.19)	0.668

[a] Separate regression models for each physical activity intensity and domain were adjusted for maternal age at enrolment, education, formal employment, parity, pre-pregnancy BMI, mode of delivery, gestational age, gestational weight gain, total energy intake during pregnancy and total physical activity during pregnancy. CI, confidence interval; GLM, general linear model; MET, metabolic equivalent of task.

Table S2. Sub-group analyses: Linear regression analyses and GLM of postpartum weight retention in relation to physical activity exposures (n=1617).

Pre-pregnancy BMI (kg/m²)[b]	Total Physical activity at 3-month postpartum (MET-hour/week)	Linear regression analyses				GLM with repeated measures	
		Postpartum weight retention at 6-month [a]		Postpartum weight retention at 12-month [a]		6-month & 12-month	
		**Coefficient (95% CI)**	***p***	**Coefficient (95% CI)**	***p***	**Coefficient (95% CI)**	***p***
Underweight (< 18.5) (n=421, 26.0%)		*p* trend = 0.640		*p* trend = 0.408		*p* trend = 0.455	
	Low (≤ 133.2)	Reference		Reference		Reference	
	Medium (> 133.2 to ≤ 163.3)	0.09 (-0.67, 0.84)	0.819	0.56 (-0.19, 1.31)	0.141	0.32 (-0.32, 0.97)	0.328
	High (> 163.3)	-0.21 (-0.99, 0.58)	0.604	-0.41 (-1.19, 0.36)	0.297	-0.31 (-0.98, 0.36)	0.368
Normal (18.5-22.9) (n=1018, 63.0%)		*p* trend = 0.0005		*p* trend = 0.0006		*p* trend = 0.0001	
	Low (≤ 133.2)	Reference		Reference		Reference	
	Medium (> 133.2 to ≤ 163.3)	-0.34 (-0.87, 0.20)	0.219	-0.26 (-0.79, 0.27)	0.334	-0.30 (-0.78, 0.18)	0.226
	High (> 163.3)	-0.99 (-1.54, -0.43)	<0.001	-0.97 (-1.52, -0.43)	0.001	-0.98 (-1.48, -0.48)	<0.001
Overweight and Obese (≥ 23.0) (n=178, 11.0%)		*p* trend = 0.007		*p* trend = 0.245		*p* trend = 0.027	
	Low (≤ 133.2)	Reference		Reference		Reference	
	Medium (> 133.2 to ≤ 163.3)	1.23 (-0.32, 2.78)	0.119	0.43 (-1.20, 2.06)	0.600	0.83 (-0.54, 2.20)	0.233
	High (> 163.3)	2.03 (0.57, 3.50)	0.007	0.89 (-0.66, 2.44)	0.257	1.46 (0.17, 2.76)	0.027

[a] Adjusted for maternal age at enrolment, education, formal employment, parity, mode of delivery, gestational age, gestational weight gain, total energy intake during pregnancy, and total physical activity during pregnancy; [b] Cut-offs from World Health Organization for Asian populations; CI, confidence interval; GLM, general linear model; MET, metabolic equivalent of task.
